# Ammonia and lactate blood levels on hospital arrival predict neurological outcome in patients with out-of-hospital cardiac arrest

**DOI:** 10.1186/cc9725

**Published:** 2011-03-11

**Authors:** K Shinozaki, S Oda, T Sadahiro, M Nakamura, Y Hirayama, E Watanabe, Y Tateishi, K Nakanishi, N Kitamura, H Hirasawa

**Affiliations:** 1 Chiba Aoba Municipal Hospital, Chiba City, Japan; 2 Graduate School of Medicine, Chiba University, Chiba City, Japan; 3 Narita Red Cross Hospital, Narita City, Japan; 4 Kimitsu Chuo Hospital, Kisarazu City, Japan

## Introduction

There is no reliable predictor on arrival at hospital for neurological outcome of the patient with out-of-hospital cardiac arrest (OHCA). We hypothesize that ammonia and lactate may predict neurological outcome.

## Methods

We performed a prospective observational study. Non-traumatic OHCA patients who gained sustained return of spontaneous circulation and were admitted to an acute care unit were included. Blood ammonia and lactate levels were measured on arrival at hospital. The patients were classified into two groups: favorable outcome group (Cerebral Performance Category (CPC) 1 to 2 at 6-month follow-up), and poor outcome group (CPC 3 to 5). Basal characteristics obtained from the Utstein template and biomarker levels were compared between these two outcome groups. Independent predictors were selected from all candidates using logistic regression analysis.

## Results

Ninety-eight patients were included. Ammonia and lactate levels in the favorable outcome group (*n *= 10) were significantly lower than those in the poor outcome group (*n *= 88) (*P *< 0.05, respectively). On receiver operating characteristic analysis, the optimal cut-off value for predicting favorable outcome was determined as 170 μg/dl ammonia, 12.0 mmol/l lactate (area under the curve: 0.714 and 0.735, respectively). Logistic regression analysis identified ammonia (≤170 μg/dl), therapeutic hypothermia and witnessed by emergency medical service personnel as independent predictors of favorable outcome. When both these biomarker levels were over threshold, the positive predictive value (PPV) for poor outcome was calculated as 100%.

## Conclusions

Ammonia and lactate blood levels on arrival are independent neurological prognostic factors for OHCA. The PPV with the combination of these biomarkers predicting poor outcome is high enough to be useful in clinical settings.

